# Virological Response and Antiretroviral Drug Resistance Emerging during Antiretroviral Therapy at Three Treatment Centers in Uganda

**DOI:** 10.1371/journal.pone.0145536

**Published:** 2015-12-23

**Authors:** Pontiano Kaleebu, Wilford Kirungi, Christine Watera, Juliet Asio, Fred Lyagoba, Tom Lutalo, Anne A. Kapaata, Faith Nanyonga, Chris M. Parry, Brian Magambo, Jamirah Nazziwa, Maria Nannyonjo, Peter Hughes, Wolfgang Hladik, Anthony Ruberantwari, Norah Namuwenge, Joshua Musinguzi, Robert Downing, Edward Katongole-Mbidde, The HIV Drug Resistance Working group

**Affiliations:** 1 Uganda Virus Research Institute, Entebbe, Uganda; 2 MRC/UVRI Uganda Research Unit on AIDS, Entebbe, Uganda; 3 London School of Hygiene and Tropical Medicine, London, United Kingdom; 4 Ministry of Health, AIDS Control Programme, Kampala, Uganda; 5 U.S. Centers for Disease Control and Prevention, Entebbe, Uganda; University of Cape Town, SOUTH AFRICA

## Abstract

**Background:**

With the scale-up of antiretroviral therapy (ART), monitoring programme performance is needed to maximize ART efficacy and limit HIV drug resistance (HIVDR).

**Methods:**

We implemented a WHO HIVDR prospective survey protocol at three treatment centers between 2012 and 2013. Data were abstracted from patient records at ART start (T1) and after 12 months (T2). Genotyping was performed in the HIV *pol* region at the two time points.

**Results:**

Of the 425 patients enrolled, at T2, 20 (4.7%) had died, 66 (15.5%) were lost to follow-up, 313 (73.6%) were still on first-line, 8 (1.9%) had switched to second-line, 17 (4.0%) had transferred out and 1 (0.2%) had stopped treatment. At T2, 272 out of 321 on first and second line (84.7%) suppressed below 1000 copies/ml and the HIV DR prevention rate was 70.1%, just within the WHO threshold of ≥70%. The proportion of participants with potential HIVDR was 20.9%, which is higher than the 18.8% based on pooled analyses from African studies. Of the 35 patients with mutations at T2, 80% had M184V/I, 65.7% Y181C, and 48.6% (54.8% excluding those not on Tenofovir) had K65R mutations. 22.9% had Thymidine Analogue Mutations (TAMs). Factors significantly associated with HIVDR prevention at T2 were: baseline viral load (VL) <100,000 copies/ml [Adjusted odds ratio (AOR) 3.13, 95% confidence interval (CI): 1.36–7.19] and facility. Independent baseline predictors for HIVDR mutations at T2 were: CD4 count <250 cells/μl (AOR 2.80, 95% CI: 1.08–7.29) and viral load ≥100,000 copies/ml (AOR 2.48, 95% CI: 1.00–6.14).

**Conclusion:**

Strengthening defaulter tracing, intensified follow-up for patients with low CD4 counts and/or high VL at ART initiation together with early treatment initiation above 250 CD4 cells/ul and adequate patient counselling would improve ART efficacy and HIVDR prevention. The high rate of K65R and TAMs could compromise second line regimens including NRTIs.

## Introduction

The 2010 guidelines recommended ART initiation for all patients with a CD4 count of ≤350 cells/mm3 and for those with WHO clinical stage 3 or 4 if CD4 testing is not available. In Uganda, applying these guidelines, the proportion of all ART-eligible patients receiving treatment was 69.4% i.e. 570,373 by the end of September 2013. However, this proportion falls to 40.0% if the 2013 WHO guidelines for ART eligibility are used [1]. The 2013 ARV guidelines recommend initiating ART earlier—at CD4 count ≤500 cells/mm3– and immediately initiating ART for sero-discordant couples, pregnant women living with HIV, people with TB and HIV, people with HIV and hepatitis B, and children living with HIV who are younger than five years, irrespective of CD4 cell count. While in well-resourced countries monitoring of people on ART is individualized and includes VL and resistance testing, in resource limited countries, WHO recommends a public health approach to ART delivery with limited laboratory monitoring [2].

Uganda’s national treatment guidelines are in line with WHO public health guidelines and comprise simplified clinical and laboratory criteria to determine ART eligibility, and standard therapeutic algorithms based on standardized first and second—line ART regimens. More recently, these guidelines have been revised [3] to reflect the new 2013 WHO guidelines. The nationally recommended treatment regimens contain 1 non-nucleoside reverse transcriptase inhibitor (NNRTI) with 2 nucleoside reverse transcriptase inhibitors (NRTIs), with a recent recommendation for TDF to be the first line drug of choice replacing AZT, partly due to its lower toxicity levels and the availability of a single pill containing TDF and FTC. Although the new guidelines also recommend VL as the preferred monitoring approach to diagnose and confirm ARV treatment failure, VL testing is not being performed in most treatment centers because of cost and limited availability. Instead CD4 count and clinical monitoring are used to diagnose treatment failure, although due to inadequate resources even the recommended CD4 counts are not always performed.

The current ART delivery in Uganda under the WHO public health approach lacks a robust system to monitor the success of the programme in suppressing VL and minimizing HIVDR. For that reason, WHO has provided guidelines for countries to implement HIV ART resistance prevention, monitoring and surveillance activities to monitor ART resistance that is transmitted among drug naïve populations, and monitor development of resistance and associated factors in those on treatment[4,5]. Following these guidelines, we have reported low to moderate transmitted drug resistance (TDR) in Uganda, 0/46 (0%), 6/70 (8.6%), 1/40 (2.5%) and 3/47 (6%), [6–9] For those on treatment, the WHO protocol is designed to use standardized, minimum-resource methodology to assess the success of adult and paediatric ART sites in preventing HIVDR emergence during the first year of ART. The surveys also identify associated factors that can be addressed at the level of the ART site or programme. Their objectives include estimation of the proportion of HIV-1 positive adults enrolled on ART achieving viral suppression after taking standard first-line ART for 12 months, identification of specific HIVDR mutation patterns among populations not achieving viral suppression after 12 months of taking standard first-line ART and to assess the association between individual and programmatic factors and viral suppression and HIVDR mutations and mutation patterns[10]. There are a number of countries that have implemented these protocols [11–16]. These surveys have been identified as the best method of identifying the emergence of HIVDR in resource-limited countries at the population level when it is impractical to look at the individual patient level. Whereas no previous studies have reported results based on the standardized WHO protocol to assess acquired drug resistance in Ugandan adults, one prospective cohort study in six African countries has reported very high rates of resistance in pre-treatment patients in Uganda probably related to the earlier ART roll out in Uganda [17]. Overall good viral suppression was reported at 12 months in the six countries [18].

With Uganda’s HIV/AIDS burden at 1.6 million according to the 2014 Ministry of Health report and with the efforts to increase the number of patients on ART including test and treat for key populations such as sex workers and fisher folk, numbers on ART are expected to exceed 1.2 million, by 2020. There were about 750,000 patients on ART by March 2015 according to Ministry of health (report in draft). With such large numbers of patients, there is an urgent need to minimize preventable HIVDR at the programme level. It is therefore critical to have prospective data in a variety of health settings to effectively monitor HIVDR and identify risk factors that might hamper success of the ART programme in Uganda. These efforts are now part of the new National HIV/AIDS strategic plan 2015/2016-2019/2020 [19].

The overall objective of our pilot study therefore was to implement the standardized WHO approach to monitoring of HIVDR emerging during ART, and associated programme factors, in three health facilities in Uganda.

## Methods

### Study design and population

A prospective cohort survey based on the WHO generic protocol [10] for monitoring acquired HIVDR was conducted at three sentinel health facilities during the period between March 2012 and November 2013. A blood specimen and minimal data were collected at baseline when ART was initiated (T1), and at follow-up i.e. 12 months after ART start and assessed for VL and HIVDR mutations (HIVDRMs). We allowed an additional 3 months to ascertain loss to follow up (LTFU). The study clinics traced patients who missed their scheduled visits mainly through phone calls. Visits to patients’ homes were also done by one of the health facility care teams when there was non-response to phone calls. This was done for patients whose residence was within 21 km radius of the health facility. Patients were classified as lost to follow-up if they didn’t attend the clinic for a scheduled appointment or drug pickup more than 90 days after the missed appointment/drug pick up and there was no information to classify them in one of the other endpoint categories of death or transfer-out. At the time of enrolment (T1), patient information was abstracted from the medical records. This included socio-demographic variables, past ART exposure; ART regimen prescribed and date of ART initiation, CD4 T-cell count and WHO clinical stage. Plasma was extracted from whole blood taken at participant enrolment, (or if not available, a freshly drawn venous sample), for VL and genotypic HIVDR testing conducted at the WHO-accredited MRC/UVRI laboratory in Entebbe. All plasma specimens at enrolment were collected on the day ART was prescribed for the patient, but before the patient consumed any dose.

A second set of non-laboratory information was collected from medical records at follow-up, (T2) i.e., either when first-line ART ended for the individual at the monitoring site, or at 12 months, if the individual was still alive and on a first-line regimen. The information included drug pick-up during the previous 12 months, changes in regimen, adherence, and clinical status at 12 months, i.e., whether the individual died, transferred out, became LTFU, stopped ART, was switched to second-line ART, or was still on first-line ART at T2. At the follow-up, we collected plasma for VL estimation, for those who switched to second-line regimen, at the time of the switch, and from those who were alive and on first-line regimen at the end of the 12 months period. Specimens with a detectable VL (plasma RNA ≥1000 copies/ml) were genotyped to detect and characterize DR mutations (DRMs).

The three sites were Masaka, Mbale regional public referral hospitals in the southern and eastern parts of the country respectively and Nsambya Home-Care ART services, an NGO hospital in Kampala. Nsambya’s support is mostly from the Presidents Emergency Plan for AIDS Relief (PEPFAR) through Catholic Relief Services.

The HIVDR local database developed by WHO and US Centres for Disease Control and Prevention Global AIDS Program was used for specimen tracking and data management.

Ethical clearance was obtained from the Uganda Virus Research Institute (UVRI) Science and Ethics Committee (SEC) and the Uganda National Council for Science and Technology (UNCST). Following written informed consent approved by SEC and UNCST, we enrolled HIV-infected adults (at least 18 years of age) eligible for ART and commencing standard first-line triple ART drug regimen for the first time. We excluded non-ART naïve individuals, i.e., adults who were taking or had previously started and stopped a standard first-line ART drug regimen. Adults exposed to ART, either during ARV prophylaxis for prevention of mother-to-child transmission or other mono or dual ART prophylactic regimens were eligible for enrolment.

### Sample size

At least 96 adults at each site with classifiable HIVDR outcome by 12 months of follow-up were required to give an estimate of the proportion of adults with HIV DR prevention at 12 months with a 95% CI of +/-10% irrespective of the incidence of HIV DR prevention at each monitoring site. We used consecutive sampling with all eligible adults for whom ART was initiated till the required sample size of 140 clients had been attained at each site (The detailed formula is under support document S1 Appendix).

### Viral Load Testing and Genotyping

Stored plasma samples obtained at enrolment and follow-up were assayed for HIV-1 RNA using the CAP/CTM (Cobas Ampliprep/Cobas Taqman 48) version 2 with a lower detection limit of 20 copies/mL. Samples with HIV-1 RNA ≥1000 copies/mL were sent for genotyping,as guided by the WHO surveillance protocol.

Viral RNA was extracted from 140ul of plasma using the QIAmp Viral RNA mini kit (Qiagen). The entire protease (codons 1–99) and amino terminus of reverse transcriptase (codons 1–320) were amplified using one-step RT-PCR kit (Qiagen); briefly 10ul extracted RNA was mixed with 6.5ul distilled water,5ul 5x PCR buffer (Invitrogen), 1ul dNTPS (Qiagen), 0.75 ul forward primer POLF-1 (5’-TGAARGAITGYACTGARAGRCAGGCTAAT-3’),0.75ul reverse primer POLR-1 (5’-CCTCITTYTTGCATAYTTYCCTGTT-3’) and 1ul enzyme mix (Qiagen). The mixture was cycled for 50°C 40 min, 95°C 15 min, [94°C 30 s, 53°C 30 s, 72°C 1min] x 35 cycles, 72°C 4 min and 10°C hold. Next 2ul of 1° PCR product was mixed with a master mix containing 36.5ul distilled water, 5ul 10x PCR buffer (Invitrogen), 2ul Mgcl_2_ (Invitrogen), 1ul dNTPs (Qiagen), 1.5ul forward primer POLF2 (5’-CTTTARYTTCCCTCARATCACTCT-3’), 1.5ul reverse primer POLR2 (5’- GGCTCTTGATAAATTTGATATGTCCAT-3’) and 0.5ul of the platinum Taq enzyme (Invitrogen) this was cycled at 95°C 5min, [94°C 30 s, 50.3°C 30 s, 72°C 1min]x 35 cycles, 72°C 2 min and 4°C hold. Gel electrophoresis was done using 1% agarose gel to ascertain the right size of the amplified PCR product. The right size PCR product was cleaned using QIAquick PCR purification kit using manufacturer’s instructions. The cleaned products were sequenced using the Big dye terminator v3.1 cycle sequencing kit (Applied Biosystems) in a reaction of 4ul of the DNA template, 6ul distilled water,2.5ul ready reaction mix, 3ul 5x SEQ buffer, 5ul of each of 3 forward primers B (5’-GTTAAACAATGGCCATTGACAGAAGA-3’), C(5’-TGGAAAGGATCACCAGCAATATTCCA-3’) and POLF2(5’-CTTTARYTTCCCTCARATCACTCT-3’) and 3 reverse primers; F (5’-GGGCCATCCATTCCTGGC-3’), G (5’-CCATCCCTGTGGAAGCACATTG-3’) and H (5’-CTGTATTTCTGCTATTAAGTCTTTTGA-3’). Cycle sequencing was performed using the following conditions 96°c for 1min [96°c for 20 s, 55°c 20 s, 60°c 4min] x 25 cycles, and 4°C hold. Sequencing was done using the ABI 3500 machine (Applied Biosystems). Sequences were base-called using Sequencher v5.2.4 and sequence alignments performed using BioEdit v7.2.5 (T. Hail, 2013) and SeaView v4.0 (Gouy M, Guindon S & Gascuel 0 2010). Quality Assurance was done using the Calibrated Population Resistance tool (CPR), Stanford and the Los Alamos National database (LANL dbase) for the HIV Sequence Quality Analysis.

DRMs classified as low, intermediate or high were assigned by submission of sequences to Stanford HIVdb Program whereas HIV-l subtypes was done using SCUEAL and REGA online software, (www.bioafrica.net/rega-genotype/html/subtypinghiv.html) and RIP (www.hiv.lanl.gov/content/seguence/RIP/RIP.htmIL). Assigned DRMs were interpreted using 2009 WHO list for epidemiological surveys alongside with the lAS 2014 Update of DRMs of HIV-1 [20]. Basic phylogenies were performed to determine sequence relatedness and to rule out contaminations.

### Statistical analyses

The outcomes of interest included baseline (T1) and follow-up (T2) factors classified according to HIVDR outcome: HIVDR prevention; possible or potential HIVDR and HIVDR as defined in the 2012 WHO protocol [5]. The following are the definitions of the above outcomes:

HIVDR prevention: The numerator includes people with viral load less than 1000 copies/ml 12 months after antiretroviral therapy initiation or at the time of switch to second-line therapy. The denominator includes people receiving first-line antiretroviral therapy at 12 months with classifiable viral load results + people switching to second-line antiretroviral therapy with classifiable viral load result + people lost to follow-up + people who stopped antiretroviral therapy during the survey.

HIV Drug Resistance: The numerator includes people with a viral load greater than 1000 copies/ml 12 months after antiretroviral therapy initiation or at switch to second-line therapy with HIV drug resistance. The denominator includes people receiving first-line antiretroviral therapy at 12 months with classifiable viral load results + people switching to second-line antiretroviral therapy with classifiable viral load result + people lost to follow-up + people who stopped antiretroviral therapy during the survey.

Possible / Potential HIV Drug Resistance: The numerator includes people with viral load greater than 1000 copies/ml and no detected HIV drug resistance at 12-month survey endpoint (on antiretroviral therapy at 12 months and at switch)+ people who stopped antiretroviral therapy + people lost to follow-up + people with unclassifiable viral load at 12-month survey endpoint (on antiretroviral therapy at 12 months and at switch). The denominator includes people on first-line antiretroviral therapy at 12 months with classifiable viral load results + people switching to second-line antiretroviral therapy with classifiable viral load result + people lost to follow-up + people who stopped antiretroviral therapy during the survey.

The above definitions can also be accessed at http://www.who.int/hiv/pub/drugresitance/report2012/en/.

All statistical analyses were performed using STATA version 12 (StataCorp LP, Texas, USA). Chi-square tests and Mann-Whitney-U tests were used to determine associations between patient characteristics for categorical variables and for continuous variables, respectively. This was done for HIVDRMs at both T1 and T2, and for viral failure (VF) (RNA viral load ≥1000 copies/ml) at T2.

VF and HIVDRMs prevalence rates with 95% confidence intervals (95% CIs) were calculated. Logistic regression was used to obtain independent predictors of HIVDR prevention, VF and HIVDRMs at T2. Variables that had a p–value of less than 0.25 in simple analysis were included in the adjusted logistic regression model. AORs, 95% CIs, and p-values were calculated.

## Results

### Study flow

**Fig 1:**
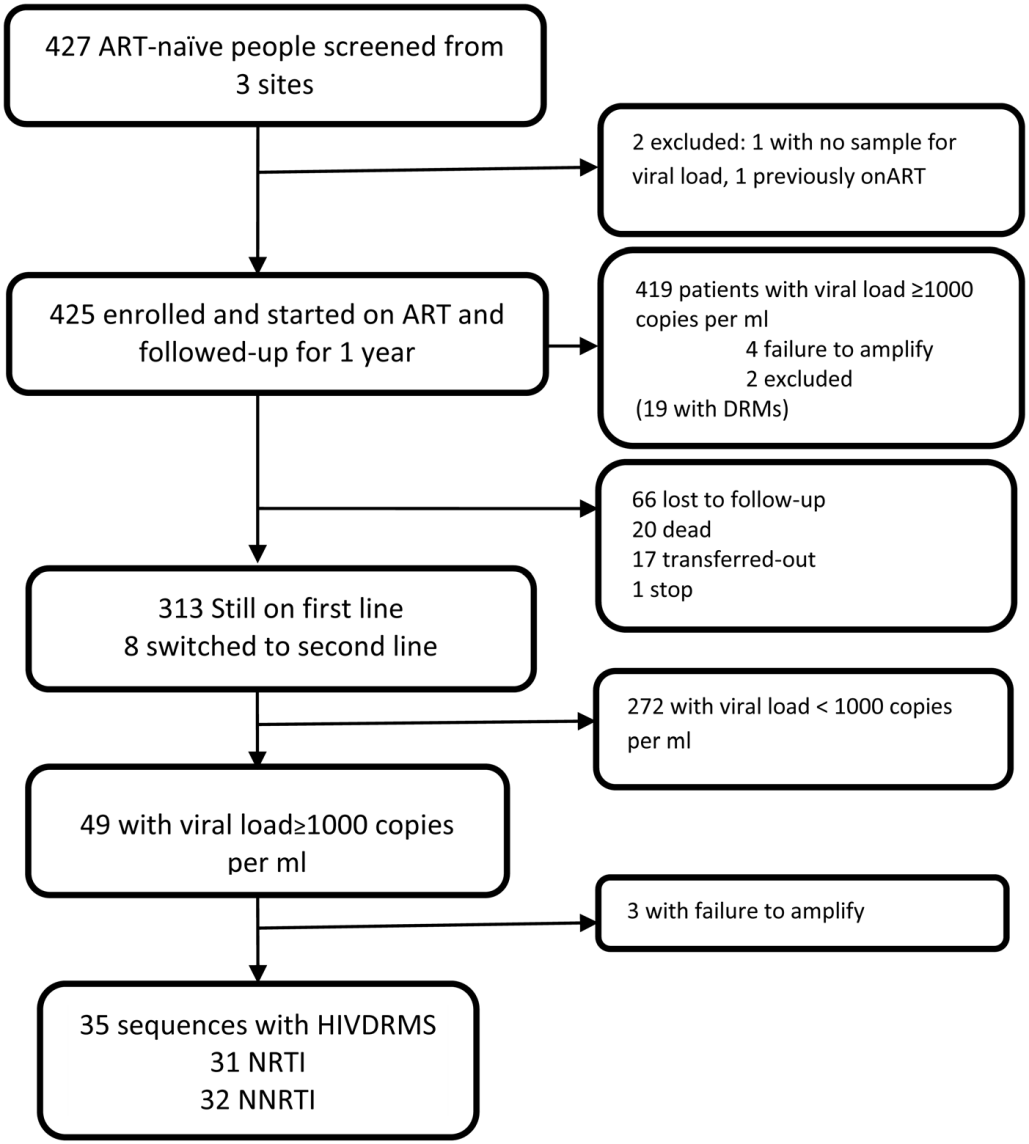
Study profile: Of the 427 participants screened, 425 were enrolled and started on ART at the three sites. Three hundred and twenty one participants completed their 12 months visit and had viral load results available at baseline and month 12. Forty nine had viral loads equal or above 1000 copies/ml and were genotyped. Of these 35 participants had HIV drug resistance mutations.

Of the 427 antiretroviral-naive patients screened, a total of 425 who met the study eligibility criteria and consented to participate in the study were consecutively enrolled, started on ART and followed-up for 1 year. Two individuals were excluded, one did not provide a sample for baseline viral load testing and another was found to have previously been on ART. Fig 1 summarises the study flow with numbers of patients who were censored for different reasons during the 1 year follow-up.

Patients who missed their scheduled visits were followed up to ascertain their status. Some of these had died or transferred to other ART treatment facilities while still on first-line regimens. However, if the status remained unknown and the patients hadn’t attended the clinic within the 2 months preceding the 12 month visit, and for another three months after the 12 month visit, they were considered lost to follow up. Patients lost to follow up contributed to the denominator in estimating the proportion of patients with the outcomes HIV DR prevention, potential HIVDR resistance and HIVDR. These patients were included in the analysis for HIV drug resistance because they would have taken some doses of ART with HIV being exposed to the ARVs and potentially having archived resistance.

### Characteristics of the study population at Baseline

Of the 425 patients from 3 sites whose specimens were sent for VL and baseline resistance testing (done when VL was ≥1000 copies): 141 (33.2%) patients were from Masaka, 143 (33.6%) from Mbale, and 141 (33.2%) from Nsambya Home Care.

Table 1 shows the characteristics of the entire study population and also compares the groups by facility. About 34% of the participants were male and the median age was 34 years. Of the 282 females in the study, 5 (1.8%) had previously received ART for PMTCT; 3 of these were from Masaka RRH (2 on TDF/FTC/EFV and 1 on TDF/FTC/NVP). Two participants were from Mbale RRH and were on TDF/3TC/NVP and AZT/3TC/NVP. None of these women exhibited HIVDRMs at baseline. There was no other form of ART exposure identified in the survey.

**Table 1:** Baseline Demographic, Clinical and Immunologic characteristics of patients receiving ART in three Sentinel Antiretroviral sites.

Characteristics	Masaka n(%)	Mbale n(%)	Nsambya n(%)	Total N(%)
**Overall**	141(100.0)	143(100.0)	141(100.0)	425(100.0)
**Gender** *				
Male	59(41.8)	41(28.7)	43(30.5)	143(33.6)
Female	82(58.2)	102(71.3)	98(69.5)	282(66.4)
**Age (years)** *				
Median (IQR)	32(28–37)	37(30–45)	33(27–40)	34(28–40)
**Education Level**				
None	14(9.9)	33(23.1)	9(6.4)	56(13.2)
Primary	72(51.1)	54(37.7)	46(32.6)	172(40.5)
Secondary	38(27.0)	37(25.9)	51(36.1)	126(29.7)
Post-Secondary	2(1.4)	6(4.2)	18(12.8)	26(6.1)
No Information	15(10.6)	13(9.1)	17(12.1)	45(10.5)
**Marital Status** *				
Single	11(7.8)	20(14.0)	31(22.0)	62(14.6)
Married	72(51.1)	87(60.8)	62(44.0)	221(52.0)
Separated	56(39.7)	34(23.8)	39(27.7)	129(30.4)
No Information	2(1.4)	2(1.4)	9(6.3)	13(3.0)
**TB Treatment**				
No	132(93.6)	136(95.1)	129(91.5)	397(93.4)
Yes	8(5.7)	4(2.8)	11(7.8)	23(5.4)
No Information	1(0.7)	3(2.1)	1(0.7)	5(1.2)
**CD4 Count (cells/μl)**				
Median (IQR)	203(81–301)	202(115–278)	208(84–314)	204(84–314)
**Baseline WHO STAGE** *				
1 or 2	119(84.4)	104(72.7)	108(76.6)	331(77.9)
3 or 4	21(14.9)	37(25.9)	25(17.7)	83(19.5)
No Information	1(0.7)	2(1.4)	8(5.7)	11(2.6)
**Subtype** ^2^ *				
A	51(37.5)	78(55.7)	63(46.0)	192(46.5)
C	6(4.4)	5(3.6)	7(5.1)	18(4.3)
D	69(50.7)	38(27.1)	52(38.0)	159(38.5)
AD	9(6.6)	19(13.6)	9(6.6)	37(9.0)
CD	1(0.8)	0(0.0)	6(4.3)	7(1.7)
**Initial ART Regimen** *				
AZT+3TC+EFV/ NVP	1(0.7)	81(56.7)	8(5.7)	90(21.2)
TDF+3TC+EFV/ NVP	4(2.8)	62(43.3)	37(26.2)	103(24.2)
TDF+FTC+EFV/ NVP	136(96.5)	0(0.0)	94(66.7)	230(54.1)
AZT/TDF+3TC+KLT	0(0.0)	0(0.0)	2(1.4)	2(0.5)
**ART exposure** ^1^ *				
No	134(95.1)	140(97.9)	128(90.8)	402(94.6)
PMTCT	3(2.1)	2(1.4)	0(0.0)	5(1.2)
No Information	4(2.8)	1(0.7)	13(9.2)	18(4.2)
**HIV DR Mutations at Baseline**				
NRTI	5(3.5)	3(2.1)	1(0.7)	9(2.1)
NNRTI	7(5.0)	4(2.8)	6(4.3)	17(4.0)
At least One	8(5.7)	5(43.5)	6(4.3)	19(4.5)
**RNA Viral Load (**x10^5^ **copies/ml)**				
Median (IQR)	1.20(0.44–3.70)	1.49(0.53–4.20)	1.28(0.37–3.42)	1.30(0.47–3.76)

* Statistically significant difference between facilities.

^1^PMTCT was the only form of ART exposure reported.

^2^only 413 samples successfully sequenced.

The median baseline CD4+ count was 204 cells/μl, and the median baseline HIV-1 plasma RNA level was 127,929 copies/ml.

Samples from 419(98.6%) patients had viral load ≥ 1000 copies/ml. Of these, 413 were successfully sequenced. HIV-1 subtype profiles were: A1 46.5% (n = 192); C 4.3% (n = 18); D 38.5% (n = 159); Recombinants 10.7% (n = 44).

All patients received standard first-line ART regimens according to the Uganda National guidelines at the time of enrolment into the survey, with 78.3% being initiated on a TDF containing regimen. Two (0.5%) patients from Nsambya HC were prescribed a ritonavir boosted lopinavir-based regimen as first-line because they had Kaposi’s sarcoma.

At baseline, drug resistance mutations (DRMs) were identified in 19(4.5%) of participants. NRTI-associated DRMs and NNRTIs-associated DRMs were found in 9 (2.1%) and 17 (4.0%) samples, respectively. Seven (1.7%) of the patients harboured mutations to more than one antiretroviral drug class, 3 (0.7%) had TAMs. None of the patients who exhibited DRMs reported a history of ART exposure. The most common NRTI- associated mutation was M184V (33.3%) out of the 15 NRTI mutations, whereas K103N and Y181C NNRTI mutations occurred at a frequency of 35.0% and 25.0% respectively (n = 20). There were three PI accessory mutations identified, two I85V and one F53L. These are unlikely to be ARV-selected and reflect natural polymorphic behaviour of protease.

### Analysis based on follow-up (T2)

The T2 characteristics of this cohort are shown in Table 2.

**Table 2:** Clinical and Virological characteristics of patients receiving ART in three Sentinel Antiretroviral sites at Follow-up.

Characteristics	Masaka n(%)	Mbale n(%)	Nsambya n(%)	Total N(%)
**Status At Endpoint**				
Dead	8(5.7)	7(4.9)	5(3.5)	20(4.7)
Lost to follow-up	23(16.3)	23(16.1)	20(14.2)	66(15.5)
Still on first line	103(73.0)	105(73.4)	105(74.5)	313(73.7)
Stop	0(0.0)	0(0.0)	1(0.7)	1(0.2)
Switch	2(1.4)	2(1.4)	4(2.8)	8(1.9)
Transfer—out	5(3.6)	6(4.2)	6(4.3)	17(4.0)
**Drug Pick-Up**				
≥ 90% On-time	35(27.3)	18(13.8)	3(2.3)	56(14.4)
**RNA Viral Load (copies/ml)**				
<1000	83(79.0)	95(88.2)	94(86.2)	272(84.7)
≥1000	22(21.0)	12(11.2)	15(13.8)	49(15.3)
**Potential HIVDR**	29(22.6)	26(20.0)	26(20.0)	81(20.9)
**HIV DR Prevention**	83(64.8)	95(73.1)	94(72.3)	272(70.1)
**HIV Drug Resistance**	16(12.5)	9(6.9)	10(7.7)	35(9.0)
**HIV DRMs at Endpoint**				
NRTI only	1(0.8)	1(0.8)	1(0.8)	3(0.8)
NNRTI only	0(0.0)	3(2.3)	1(0.8)	4(1.0)
Both NRTI & NNRTI	15(11.7)	5(3.8)	8(6.2)	28(7.2)

Out of the 425 patients enrolled, 20 (4.7%) patients had died, 66 (15.5%) were LTFU, 313 (73. 7%) were still on first-line, 8 (1.9%) had switched, 17 (4.0%) had transferred out (on first-line regimen) and 1 (0.2%) stopped treatment.

Retention in care (patients who were still on first-line, switch and transferred-out) was 79.6% which was within the WHO threshold of 75–85% [16]. Only 56 (14.4%) patients picked up their drugs on time, for at least 90% of the scheduled times during the 12 months follow-up period. Masaka registered the highest number of patients who picked up their drugs on time (p<0.001).

Of the 321 patients retained at 12 months, the median CD4 cell count was 325 cells/μl (IQR: 200–490), which increased significantly from the median of 204 cells/μl (IQR: 92–292) at baseline (P<0.0001).

At T2, out of the 321 patients with VL results, 272 (84.7%) suppressed below 1,000 copies/ml. However, using a denominator of 388 patients (which includes the patients still on first line = 313, those LTFU = 66 and those who switched to second-line regimen = 8), the overall HIV DR prevention rate was 272/388 (70.1%) due to LTFU and this was just within the recommended WHO target of ≥70%. At individual clinics these were 64.8% at Masaka, 73.1% at Mbale and 72.3% at Nsambya (p = 0.28).

The proportion of participants with potential or possible HIVDR was 20.9% (81/388) which is higher than the 18.8% based on pooled analyses from African studies [15] At individual clinics these were, 22.6% at Masaka, 20.0% at Mbale and 20.0% at Nsambya (p = 0.22).

At T2, of the 19 patients with HIV DRMs at baseline: 1 died before the one year end point, 8 were LTFU, 3 were switched to second-line regimen, 2 were transferred out and 5 were still on first-line regimen. After the 12 months of follow-up, all the three patients who were switched to second-line regimen and only 1 out of the 5 who were still on first-line regimen achieved VL suppression. The 4 patients who did not achieve VL suppression at follow-up, had HIVDRMs and three of them accumulated additional mutations (data not shown).

The proportion of participants with HIVDR at T2 was 35/388 (9.0%), which is higher than the 4.7% reported in the African region based on pooled analyses^15^. At individual sites, these were 12.5% in Masaka, 6.9% in Mbale and 7.7% from Nsambya (Table 2). There was no significant difference in the prevalence of mutations between sites at follow—up (p = 0.24).

Out of the 49 virologically failing therapy (≥1000 copies/ml) at 12 months, 35 (71.4%) had HIVDRM, comparable to the 69.5% based on pooled analyses from Africa [16]

Of the 35 patients with mutations at T2, 80% had M184V/I, 65.7% Y181C and 48.6% (54.8%, if those not on Tenofovir are excluded) had K65R mutations. There were 8/35 individuals with TAMs (22.9%), (data not shown).

### Predictors for HIV Drug Resistance Prevention and Mutations

The factors that were significantly associated with HIVDR prevention at 12 months were: VL < 100,000 copies/ml at baseline (AOR 3.13, 95% CI: 1.36–7.19) and facility, with lower HIVDR prevention at the Masaka site. (Table 3).

**Table 3:** Association between baseline patient characteristics and HIVDR Prevention at 12 Months after ART Initiation.

Characteristic	HIVDR prevention** (%)	Unadjusted OR (95% CI)	*P-Value*	Adjusted R*** (95% CI)	*P*-Value
**Gender**					
Male	110(84.6)	1		1	
Female	229(88.8)	1.43(0.78–2.65)	0.25	1.17(0.59–2.37)	0.64
**Age (years)**					
≤ 30	123(89.1)	1		1	
31–39	115(84.6)	0.67(0.33–1.36)	0.27	1.01(0.46–2.22)	0.98
≥40	99(89.2)	1.01(0.45–2.24)	0.98	1.38(0.55–3.43)	0.49
**Education Level**					
None	43(86.0)	1			
Primary	136(87.7)	1.17(0.46–2.96)	0.75		
Secondary	101(88.6)	1.26(0.47–3.39)	0.64		
Post-Secondary	24(92.3)	1.95(0.38–10.16)	0.43		
No Information	35(81.4)	0.71(0.23–2.16)	0.55		
**Marital Status**					
Single	48(87.3)	1			
Married	185(89.4)	1.22(0.49–3.04)	0.66		
Separated*	96(84.2)	0.78(0.30–1.99)	0.60		
No information	10(83.3)	0.73(1.13–4.04)	0.72		
**Facility**					
Masaka RRH	106(82.8)	1		1	
Mbale RRH	118(90.8)	2.04(0.96–4.32)	0.06	2.73(1.22–6.36)	0.02
Nsambya	115(88.5)	1.59(0.78–3.23)	0.12	2.32(1.04–5.20)	0.04
**TB Treatment**					
No	319(87.6)	1			
Yes	16(84.2)	0.75(0.21–2.68)	0.67		
**Adherence Group**					
No	11(84.6)	1			
Yes	321(87.7)	1.30(0.28–6.04)	0.74		
**CD4 Count (cells/μl)**					
<250	197(84.2)	1		1	
≥250	138(92.6)	2.35(1.16–4.78)	0.02	1.81(0.82–4.00)	0.14
**WHO Stage**					
3 and 4	57(80.3)	1		1	
1 and 2	274(89.3)	2.04(1.03–4.05)	0.04	1.49(0.71–3.18)	0.29
**HIV Subtype**					
A	151(86.3)	1			
C	14(87.5)	1.11(0.24–5.20)	0.89		
D	126(88.1)	1.17(0.61–2.29)	0.63		
Recombinants	35(85.4)	0.93(0.35–2.44)	0.87		
**Drug Pick-up**					
Not On-time	287(86.5)	1		1	
On-time	52(92.9)	2.04(0.70–5.91)	0.19	2.38(0.77–7.24)	0.14
**ART Regimen**					
AZT+3TC+EFV/NVP	75(92.6)	1			
TDF+3TC+EFV/NVP	84(90.3)	0.75(0.25–2.19)	0.59		
TDF+FTC+EFV/NVP	179(84.4)	0.43(0.17–1.08)	0.72		
**Viral load (Copies/ml)**					
≥100,000	164(94.2)	1	<0.001	1	0.01
<100,000	174(81.7)	3.68(1.78–7.68)		3.13(1.36–7.19)	

*Either separated, divorced or widowed

** HIVDR prevention includes <1000 vl + LTFU + Stopped

*** only variables with p-value <0.25 and a priori confounders were included in this analysis

Independent predictors for HIVDRMs at 12 month were: CD4 count at baseline <250 cells/μl (AOR 2.80, 95% CI: 1.08–7.29) and baseline VL ≥100,000 RNA copies/ml had a borderline association, (AOR 2.48, 95% CI: 1.00–6.14).(Table 4).

**Table 4:** Association between baseline patient characteristics and HIV Drug Resistance Mutations at 12 Months after ART Initiation.

Characteristic	Mutations n(%)	Unadjusted OR (95% CI)	*P*-Value	Adjusted OR** (95% CI)	*P*-Value
**Gender**					
Male	14(10.8)	1		1	
Female	21(8.1)	0.73(0.36–1.50)	0.39	0.92(0.42–2.03)	0.84
**Age (years)**					
≤ 30	14(10.1)	1		1	
31–39	11(8.1)	0.78(0.34–1.78)		0.51(0.21–1.27)	0.15
≥40	10(9.0)	0.88(0.37–2.06)	0.84	0.64(0.24–1.69)	0.37
**Education Level**					
None	5(10.0)	1			
Primary	13(8.4)	1.21(041–3.59)	0.73		
Secondary	9(7.9)	1.30(0.41–4.08)	0.66		
Post-Secondary	2(7.7)	1.33(0.19–7.39)	0.74		
No Information	6(14.0)	0.69(0.19–2.43)	0.56		
**Marital Status**					
Single	5(9.1)	1			
Married	16(7.7)	1.19(0.42–3.41)	0.74		
Separated*	13(11.4)	0.78(0.26–2.30)	0.65		
Unknown	1(8.33)	1.1(0.11–10.4)	0.93		
**Facility**					
Masaka RRH	16(12.5)	1		1	
Mbale RRH	9(6.9)	0.52(0.22–1.22)	0.14	0.43(0.17–1.08)	0.07
Nsambya	10(7.7)	0.58(0.25–1.34)	0.20	0.52(0.21–1.24)	0.14
**TB Treatment**					
No	33(9.1)	1			
Yes	2(10.5)	1.18(0.26–5.33)	0.83		
**CD4 Count (cells/μl)**					
≥250	6(4.0)	1		1	
<250	29(12.4)	3.37(1.36–8.33)	0.01	2.80(1.08–7.29)	0.04
**WHO Stage**					
1 and 2	24(7.8)	1		1	
3 and 4	11(15.5)	2.16(1.05–4.65)	0.04	1.62(0.71–3.74)	0.23
**HIV Subtype**					
A	17(9.7)	1			
C	2(12.5)	1.33(0.28–6.81)	0.72		
D	13(9.1)	0.93(0.44–1.98)	0.85		
Recombinants	3(7.3)	0.73(0.20–0.26)	0.64		
**Drug Pick-up**					
Not On-time	31(9.3)	1			
On-time	4(7.1)	0.75(0.25–2.20)	0.59		
**Initial ART Regimen**					
AZT+3TC+EFV/NVP	4(4.9)	1			
TDF+3TC+EFV/NVP	8(8.6)	1.81(0.52–6.26)	0.35		
TDF+FTC+EFV/NVP	23(10.9)	2.34(0.78–6.70)	0.13		
**Viral load (Copies/ml)**					
<100,000	8(4.6)	1		1	
≥100,000	27(12.7)	3.01(1.33–6.77)	0.01	2.48(1.00–6.14)	0.05

*Either separated, divorced or widowed

** only variables with p-value <0.25 and a priori confounders were included in this analysis

Considering only patients with VL results at follow-up, the factors that were significantly associated with VF were: Facility, with higher VF at Masaka and base line VL ≥ 100,000 copies/ml (AOR 3.09, 95% CI: 1.34–7.11),; there was a weak association between VF and CD4 count at baseline <250 cells/ul (AOR 2.00, 95% CI: 0.91–4.35)and patients who picked up drugs late (AOR 2.78, 95% CI: 0.86–9.00).(Table 5).

**Table 5:** Association between baseline patient characteristics and Virological failure at 12 Months after ART Initiation

Characteristic	VL ≥1000** Copies/ml (%)	Unadjusted OR (95% CI)	*P*-Value	Adjusted OR** (95% CI)	*P*-Value
**Gender**					
Male	20(18.2)	1		1	
Female	29(13.8)	0.73(0.39–1.36)	0.32	0.93(0.45–1.92)	0.85
**Age (years)**					
≤ 30	15(13.8)	1		1	
31–39	21(18.3)	1.40(0.68–2.88)	0.36	1.01(0.45–2.26)	0.98
≥40	12(12.8)	0.92(0.41–2.17)	0.84	0.73(0.29–1.88)	0.51
**Education Level**					
None	7(18.4)	1			
Primary	19(14.1)	0.72(0.28–1.88)	0.51		
Secondary	13(13.8)	0.71(0.26–1.95)	0.51		
Post-Secondary	2(9.5)	0.47(0.09–2.48)	0.37		
Unknown	8(24.2)	1.42(0.45–4.44)	0.55		
**Marital Status**					
Single	7(16.3)	1			
Married	22(12.6)	0.74(0.29–1.87)	0.52		
Separated*	18(19.0)	1.20(0.46–3.13)	0.71		
Unknown	2(25.0)	1.71(0.29–10.3)	0.56		
**Facility**					
Masaka RRH	22(21.0)	1		1	
Mbale RRH	12(11.2)	0.47(0.22–1.02)	0.06	0.37(0.16–0.85)	0.02
Nsambya	15(13.8)	0.60(0.29–1.24)	0.17	0.39(0.17–0.88)	0.03
**TB Treatment**					
No	45(15.0)	1			
Yes	3(20.0)	1.42(0.39–5.24)	0.60		
**Adherence Group**					
No	2(18.2)	1			
Yes	45(14.9)	0.78(0.16–3.75)	0.76		
**CD4 Count (cells/μl)**					
≥250	11(8.9)	1		1	
<250	37(19.2)	2.43(1.19–5.00)	0.02	2.00(0.91–4.35)	0.08
**WHO STAGE**					
1 and 2	33(12.8)	1		1	
3 and 4	14(26.4)	2.45(1.20–4.99)	0.02	1.18(0.80–3.89)	0.12
**HIV Subtype**					
A	24(16.2)	1			
C	2(20.0)	1.29(0.25–6.46)	0.76		
D	17(14.1)	0.84(0.43–1.65)	0.62		
Recombinants	6(18.6)	1.19(0.44–3.21)	0.72		
**Drug Pick-up**					
On-time	4(8.5)	1		1	
Not On-time	45(16.4)	2.11(0.72–6.18)	0.14	2.78(0.86–9.00)	0.09
**ART Regimen**					
AZT+3TC+EFV/NVP	6(9.23)	1			
TDF+3TC+EFV/NVP	18(17.1)	2.03(0.76–5.42)	0.12		
TDF+FTC+EFV/NVP	23(15.8)	1.84(0.04–0.24)	0.21		
**Viral load (Copies/ml)**					
<100,000	10(20.4)	1		1	0.01
≥100,000	39(79.6)	3.68(1.76–7.66)	0.001	3.09(1.34–7.11)	

*Either separated, divorced or widowed

** only variables with p-value <0.25 and a priori confounders were included in this analysis

## Discussion

This report, the first in Uganda to use a standardized WHO protocol to assess acquired drug resistance, provides information on the performance of the treatment programmes at three sites and associated factors, effectiveness of the current regimens and implications for second-line in a programme setting. The study was also undertaken after the introduction of new treatment guidelines with TDF as first drug of choice, prescribed to nearly 80% of the study participants.

We followed 425 patients from the three centers; these initiated treatment at relatively low CD4 counts median of 204 cells/ul, a common observation in low income countries [12,14,21,22]. The dominance of females initiating ART at all these sites possibly reflects the general observed higher access to care by females that has previously been reported[23] and the higher numbers of women infected in Uganda. The majority of patients were initiated on the nationally recommended treatment regimens containing 1 NNRTI + 2 NRTIs whereas two patients were started on PI-based regimen because they had Kaposis’s sarcoma. The HIV-1 subtypes observed reflect what has been reported before in these different parts of Uganda [7,8,24]

About 4.5% of ART-naïve subjects initiating ART and with no reported prior ART exposure had resistance mutations. This is slightly less than the 5.0% reported from low and middle income countries [16] At baseline, NNRTI mutations were the most frequent at 4.0% followed by NRTI at 2.1%. There were no PI mutations observed in this survey at baseline while 1.6% had DRMs to more than one ART class. Three individuals had TAMs, known to lead to resistance to multiple NRTIs. The presence of individuals with multiclass resistance and TAMs may be a reflection of non-disclosed ART exposure, which is a challenge to ART roll-out since it affects ART efficacy, although TDR is a possibility in some of these individuals.

There was good retention in care comparable to other studies, with 79.5% of patients retained in care, which is above the WHO-required threshold of 70%; [16] all three sites met this threshold. Our study showed an overall LTFU rate of 15.3%; WHO early warning indicator guidance recommends that no more than 20% of patients should be LTFU 12 months after treatment initiation.

Although 84.7% of participants with VL measurements at follow-up suppressed below 1000 copies/ml (Table 2), the overall HIVDR prevention rate was lower at 70.1% due to LTFU just within the recommended WHO target of ≥70%. At individual clinics, the Masaka site was below this WHO threshold at 64.8%.

The above results coupled with the good CD4 recovery indicate that those who are retained in care do well in terms of HIVDR prevention and immunological recovery; however at the programme level HIVDR prevention is lower probably due to the LTFU.

At 12 months, overall 20.9% (Table 2) were classified as potential or possible HIVDR; this is higher than 18.8% from a pooled analyses reported in the African region[16]. Our high rates could again be largely due to the reported rates of LTFU at 15.3%. These individuals are more likely to be non-adherent leading to DRMs.

The proportion of participants with HIVDRM at T2 was 9.0%(35/388), higher than the 4.7% reported in the African region based on pooled analyses of 239 patients failing therapy at 12 months [16] and two recent studies in Cameroon and Namibia that reported HIVDR prevalence of 5.3% and 5% respectively[14,15].

At T2, 71.4% (35/49)of the individuals virologically failing therapy had DRMs (Table 2) very much comparable to the average pooled analyses from Africa [16]. This indicates that most of the failure is due to resistance and a smaller proportion probably due to very low adherence.

The resistance patterns seen in our study have been reported before in patients started on these first-line regimen containing 3TC and FTC NRTIs and EFV, NVP NNRTIs [16,25]. K65R is now emerging following the recent introduction of TDF as a first-line drug in this subtype A and D infected population. K65R previously reported to be associated more with subtype C is a mutation associated with cross resistance to NRTI other than TDF and hence has the potential to compromise NRTI backbones in the second-line. On the other hand the K65R and the M184V increase AZT susceptibility which is one of the recommended second-line NRTI. The Uganda guidelines recommend as second-line ART in adults 2 NRTIs and a ritonavir boosted PI. After failure on TDF + 3TC, use AZT+3TC; after failure on AZT+3TC, use TDF + 3TC or ABC + 3TC. Boosted Atazanavir (ATV/r) is the preferred PI option for second-line ART. There were (22.9%) 8/35 individuals with TAMs (data not shown), higher than in a pooled analyses from 269 patients, where one or more TAMs were identified at 12 months in 15.6% of people being treated[16]. Again though TAMs are associated with reduced susceptibility to a number of NRTIs, the second-line regimens in the above guidelines would be effective.

In our study, factors associated with HIVDR prevention at 12 months were baseline VL < 100,000 copies/ml and facility. Low baseline CD4 counts <250 and high VL ≥ 100,000 copies/ml were associated with presence of HIVDRMs at 12 months. Considering only patients with VL results at endpoint, the factors that were significantly associated with VF were baseline VL ≥ 100,000 copies/ml and facility. There was also a weak association between VF and CD4 count at baseline <250 cells/μl and patients who picked up drugs late.

The lower HIVDR prevention and higher VF in Masaka could be due to insufficient pre-ART counselling provided by few counsellors per patients compared to the other two sites. Masaka had three counsellors supporting an average of 100 new ART patients per month compared to Mbale’s five counsellors for an average of 43 new ART patients and Nsambya’s seven counsellors for an average of 50 new ART patients. The patient’s comprehension of the importance of adherence and overall readiness for ART is likely to be compromised and hence poorer outcome for Masaka. Furthermore, this clinic engaged an additional cadre of lay health workers as adherence counsellors to assist in providing ART specific counselling. Much as these health workers received some basic training, the course was not accredited by the Ministry of Health AIDS Control Programme, hence questionable quality.

To improve treatment outcomes and prevent HIVDR emergence, there is therefore a need to enrol patients into care early enough and to have facility targeted interventions such as adequate pre-ART counselling. WHO has recently revised the treatment guidelines recommending starting treatment in adults and adolescents with HIV at CD4 cut-off of <500 cells/mm3 regardless of clinical stage, Uganda adopted these in early 2013, however patients continue to enrol at low CD4 counts.

The only information we could collect on adherence at all facilities was whether or not a patient had received pre-ART counselling recorded as adherence group and this did not affect out-come measures, although the non-group numbers were very few.

Adherence assessment based on physical counts of pills remaining at the time patients attend clinic for refill, and self-recall of missed doses during the previous month should be recorded in the patient’s clinical cards at every visit. However, this was not always done. This assessment was done at all visits for 16% and 58% of patients in Masaka and Nsambya sites respectively and the majority (>90%) had good (of ≥95%) adherence. Mbale data was unavailable because the facility used a manual register unlike the other two which used electronic medical registers making it difficult to summarise data for all patients’ visits.

Gender, age at ART initiation, education level, marital status, PMTCT exposure, viral subtype and treatment regimens did not affect 12 months HIVDR prevention and HIVDR mutation outcome.

Some of the limitations of the study include the non-generalizability of these results which are more site specific rather than national or regional. In addition, 55 participants with low vireamia 50-<1000 copies/ml were not genotyped, the WHO protocol excludes these yet there could be HIVDR in these individuals as well.

## Conclusions

There was good retention in care and a high percentage of these individuals had suppressed VLs below 1000 copies/ml (84.7%). The overall HIV DR prevention rate at programme level was just within the recommended WHO target of ≥70%, and it was below this threshold at one clinic. The proportion of participants with potential HIVDR (20.9%) and the proportion with HIVDRM at 12 months (9.0%) were also overall higher than the average from other African sites. This performance could be due to the LTFU and hence the need to strengthen defaulter tracing to improve treatment outcome.

The second-line regimens recommended were appropriate, however, the high rate of the K65R and TAMs is likely to compromise second-line drugs containing other NRTI backbone. More advanced disease at ART initiation was associated with lack of suppression and HIVDR. Efforts to initiate patients on treatment above CD4 250 cells/ul would contribute to improved ART efficacy and HIVDR prevention.

## Supporting Information

S1 Appendix This is the formula for calculating sample size. (DOCX)
