# Supplementary material for: Virological Response and Antiretroviral Drug Resistance Emerging during Antiretroviral Therapy at Three Treatment Centers in Uganda
Source: PLoS One. 2015 Dec 23;10(12):e0145536. doi: 10.1371/journal.pone.0145536 (PMC4689474; doi:10.1371/journal.pone.0145536)
Supplement: S1 Appendix — (DOCX) [file pone.0145536.s001.docx]

**S1 Appendix**

**Formula for Calculating Sample size**

At least 96 adults with classifiable HIVDR outcome by 12 months of follow-up were required to give an estimate of the proportion of adults with viral suppression at 12 months and a 95% confidence interval of +/-10% irrespective of the incidence of viral suppression at each monitoring site. Estimating a prevalence of the outcome of 50% provided the biggest sample size determination for a given precision. This was obtained based on the formula for sample size assuming a Normal distribution and ignoring the finite population correction i.e.:

*z^2^ * (p * (1 - p))
n = -------------------
           e^2^*

To estimate an unknown proportion within 0.1 (i.e. 10%) of the true population proportion with a probability of 95% (i.e. *α* = 0.05):

1.96^2^ * (0.5 * (1 - 0.5))
n = --------------------------
              0.1^2^

3.8416 * 0.25
n = -------------
        0.01

n = 96

The formula without the finite population correction, provided a sufficient survey sample size regardless of clinic size. Additional clients were enrolled to take into account the numbers that were likely to transfer out, die or get lost to follow-up during 12 months of follow-up, which was approximately 30% of clients. Therefore, 138 adults, (rounded off) to 140 clients were enrolled at each monitoring site.
